# Arachnoiditis Ossificans

**DOI:** 10.5334/jbsr.2458

**Published:** 2021-06-17

**Authors:** Clara Brou, Marie-Anne Labaisse, Patrice Jissendi Tchofo

**Affiliations:** 1 CHwapi, BE; 2 CHU Saint-Pierre, BE

**Keywords:** arachnoiditis ossificans, spinal MRI/CT, spine surgery

## Abstract

This case shows the MRI and CT features of a rare entity, namely arachnoiditis ossificans, which should be recognized in patients with long-standing history of multiple spine surgery.

## Case Report

**Figure 1:**
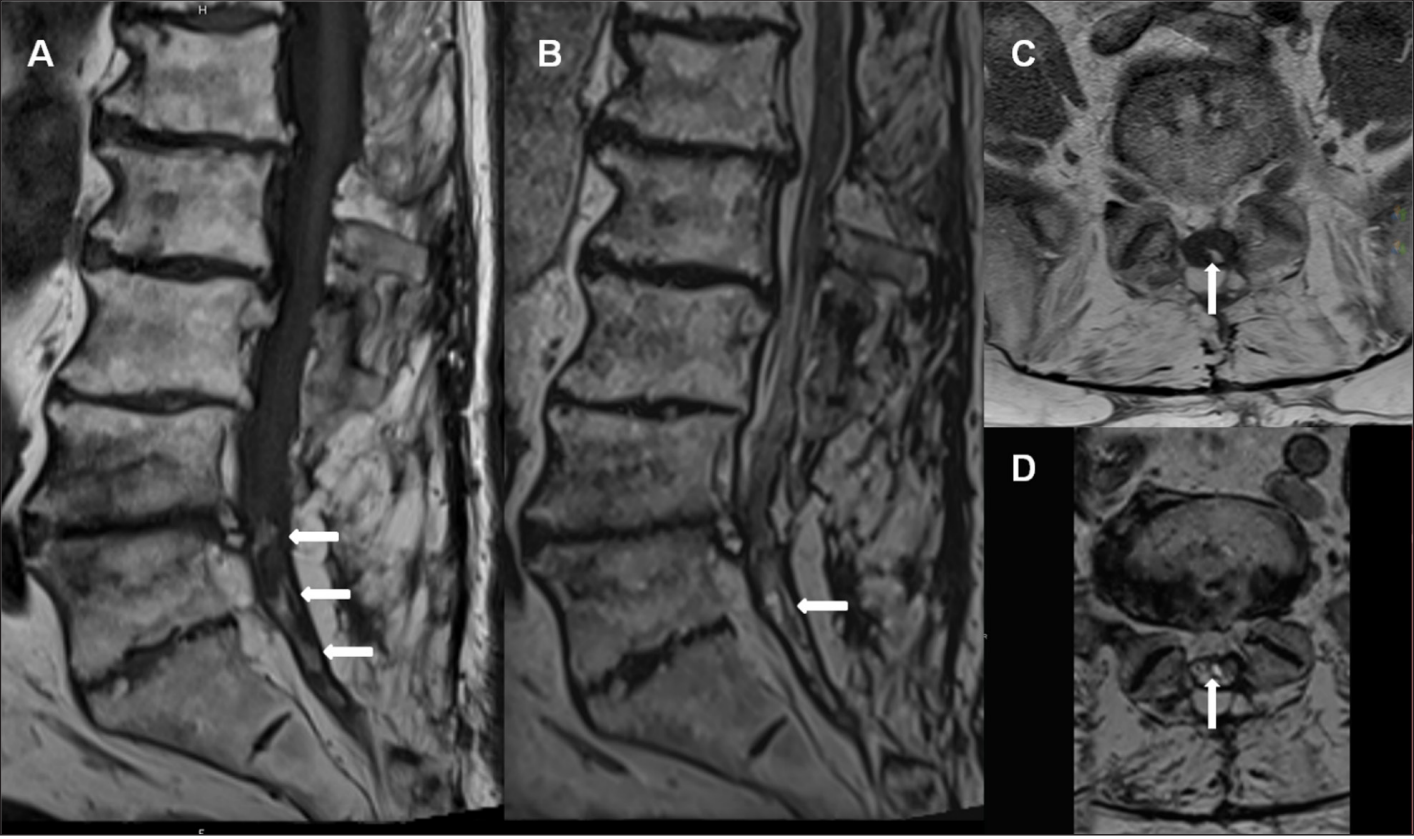


**Figure 2:**
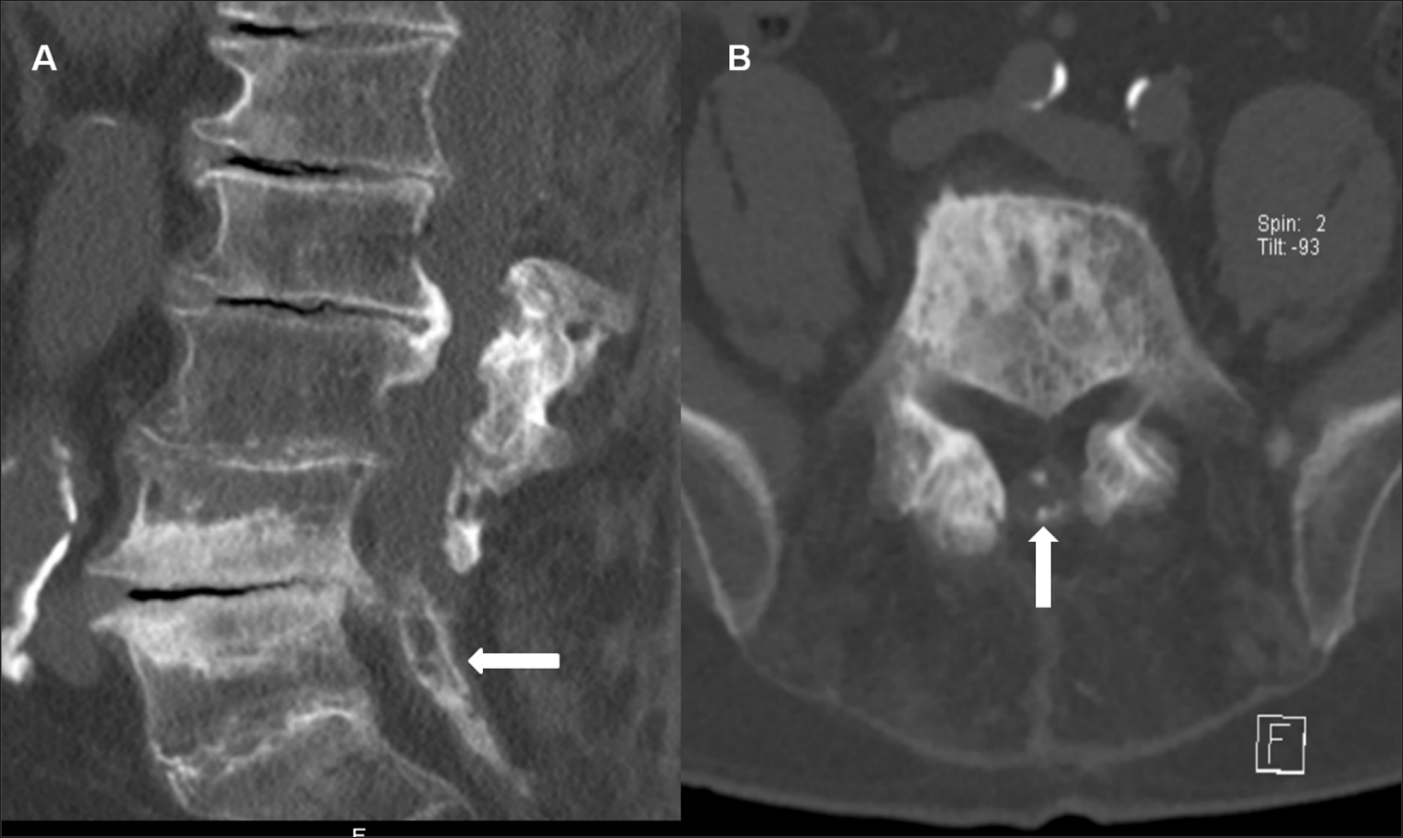


We report the case of a 77-year-old man who presented with long-standing back pain and paraparesis, but normal reflexes and no sphincter disturbances. His medical records include multiple operations of the spine with multi-level laminectomy. He experienced increased walking and balance disorders, for which he underwent a lumbar spine magnetic resonance imaging (MRI). It revealed multi-level discarthrosis and diffuse thickening of the terminal thecal sac with aggregated nerve roots, likely resulting from previous arachnoiditis. Within the canal some hyperintense T1 (***Figure 1A, 1C***, arrows) areas with hypointense T2 (***Figure 1B, 1D***, arrows) rims were found, suggesting calcifications, ossification, or hemosiderin deposits. In order to better differentiate between ossification and hemosiderin, computed tomography (CT) was performed. The hypo- T2 and hyper- T1 signals corresponded to bony densities (***Figure 2A, 2B***, arrows), so MRI and CT features were consistent with arachnoiditis ossificans (AO).

## Comment

Small areas of calcified leptomeninges that develop with aging are commonly found at surgery or autopsy. These asymptomatic calcifications have to be distinguished from intrathecal ossification resulting from chronic arachnoiditis and known as “arachnoiditis ossificans”, which is a rare condition characterized by ossification within the arachnoid. The physiopathology of the disease is uncertain. Osseous metaplasia may occur secondary to any process that maintains chronic inflammation. Nevertheless, other causative mechanisms have been described: intradural hematoma that ossifies further, or seeded bone fragments. Other etiologies include previous multiple surgeries (as in our patient), trauma, arachnoid hemorrhage, intrathecal drug injection, and spinal infections. Symptoms are variable, up to severe disability, regardless of the extent of ossification. AO may also affect other spinal segments, most often the thoracic and rarely the cervical. The differential diagnosis includes ossification of the posterior spinal ligaments, retained Pantopaque or Lipiodol, intrathecal primary neoplasm or metastases, calcified dural plaques or hemosiderin. CT and MRI are complementary for the diagnosis, as shown in our case. MRI shows irregular thickening and clumping of nerve roots of the cauda equina, while CT is useful to confirm the diagnosis by showing the mineral component. Various patterns of AO were reported by Domenicucci and classified as: Type I, at the thoracic level, as a semicircular pattern of ossification that involves part of the thecal sac; Type II, at either the thoracic or lumbar level, as a circular pattern that involves the circumference of the thecal sac; and Type III, exclusively at the lumbar level, with ossification of the entire content of the thecal sac [1]. Currently there is no consensus on either a surgical or conservative treatment for AO. Surgery remains controversial because of the risk for worsening the patient’s condition.

## References

[1] Domenicucci M, Ramieri A, Passacantilli E, Russo N, Trasimeni G, Delfini R. Spinal arachnoiditis ossificans: Report of three cases. Neurosurgery. 2004; 55: 985. DOI: 10.1227/01.NEU.0000137281.65551.54 15934184

